# Harmony-based data integration for distributed single-cell multi-omics data

**DOI:** 10.1371/journal.pcbi.1013526

**Published:** 2025-09-30

**Authors:** Ruizhi Yuan, Ziqi Rong, Haoran Hu, Tianhao Liu, Shiyue Tao, Wei Chen, Lu Tang

**Affiliations:** 1 Department of Biostatistics and Health Data Science, University of Pittsburgh, Pittsburgh, Pennsylvania, United States of America; 2 Department of Pediatrics, University of Pittsburgh, Pittsburgh, Pennsylvania, United States of America; University of Western Ontario: Western University, CANADA

## Abstract

Large-scale single-cell projects generate rapidly growing datasets, but downstream analysis is often confounded by data sources, requiring data integration methods to do correction. Existing data integration methods typically require data centralization, raising privacy and security concerns. Here, we introduce Federated Harmony, a novel method combining properties of federated learning with Harmony algorithm to integrate decentralized omics data. This approach preserves privacy by avoiding raw data sharing while maintaining integration performance comparable to Harmony. Experiments on various types of single-cell data showcase superior results, highlighting a novel data integration approach for distributed multi-omics data without compromising data privacy or analytical performance.

## Author summary

In recent years, single-cell technologies have allowed scientists to study individual cells in great detail, helping us understand how tissues and organs function. As researchers around the world generate more data, combining these datasets has become important—but also challenging. One major issue is that data from different sources often vary due to technical differences, making integration tricky. Another growing concern is privacy: many institutions are hesitant to share sensitive biological data due to ethical, legal, and security risks. In our study, we introduce a method called Federated Harmony, which allows researchers to combine single-cell data from different institutions without directly sharing any raw data. By adapting a widely used data integration method (Harmony) into a federated learning framework, our approach preserves privacy while achieving comparable scientific results. We tested Federated Harmony on several types of single-cell data and found that it performs just as well as existing centralized methods, but with improved speed and security. We believe this method could help research teams around the world collaborate more safely and effectively, accelerating discoveries in biology and medicine.

## Introduction

With the recent advances in single-cell technology [1], projects like Human Cell Atlas [2] are generating a rapidly growing collection of reference datasets from primary human tissues at different institutions, and these datasets significantly enhance our understanding of single-cell mechanisms. However, genuine biological signals in those datasets from different studies are often confounded by data source [3], which can significantly influence the downstream analysis.

Existing data integration methods [4–9] address above challenges by mapping cells from various experimental conditions and biological contexts into a unified, lower-dimensional space, facilitating the downstream analysis across different datasets. However, these methods typically require data centralization, which raises significant privacy and security concerns [10]. While policies such as the NIH Genomic Data Sharing Policy [11] mandate the sharing of transcriptomics and chromatin-related data, including raw files, to ensure reproducibility and foster collaboration, data sharing remains complex and constrained due to several additional challenges. Cross-border regulations, such as GDPR in Europe, PIPL in China, and LGPD in Brazil [12–15], impose strict limitations on genomic data transfer, creating legal barriers even when U.S. policies allow sharing. Furthermore, institutional privacy policies, ethical considerations—particularly for Indigenous and sensitive populations—and concerns over intellectual property and data ownership can further restrict data access. Additionally, cybersecurity risks associated with centralized repositories increase concerns over potential data breaches and re-identification threats [16]. Computational bottlenecks and storage limitations also make large-scale single-cell data centralization impractical, particularly for institutions with limited infrastructure under privacy regulation. These regulatory, ethical, and technical barriers create a fundamental conflict between the growing need to integrate rapidly expanding single-cell datasets and the constraints on data sharing. Thus, privacy-preserving methods are crucial for enabling secure, scalable, and globally collaborative single-cell research while ensuring compliance with diverse legal and institutional policies.

To address above challenges, we proposed Federated Harmony, a privacy-preserving method that combines federated learning and Harmony. Federated learning is a collaborative paradigm that enables institutions to collaboratively train models without raw data sharing [17], addressing privacy concerns [18–20] and reducing computational strain [21]. In addition, Harmony has also been proven as one of the best performing and most commonly used data integration methods in single-cell data analysis [22,23]. The basic idea of Federated Harmony is by incorporating Harmony into the federated learning framework. By performing local computations and sharing only aggregate statistics based on the Harmony algorithms, Federated Harmony addresses privacy concerns and reduces the computation time while maintaining integration performance comparable to Harmony. We evaluate Federated Harmony on scRNA-seq, scATAC-seq and spatial transcriptomics data, and both visual and quantitative results are promising, highlighting Federated Harmony as a promising approach for secure and efficient distributed single-cell data integration.

## Results and discussion

**Fig 1:**
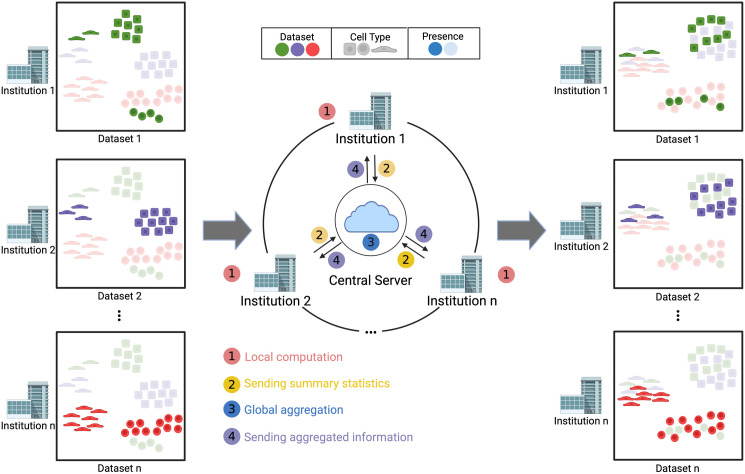
The Workflow of Federated Harmony. Federated Harmony operates through an iterative four-step process: ① Institutions perform local computations on their data; ② Summary statistics are sent to a central server; ③ The central server aggregates and updates the summaries; and ④ Aggregated summaries are returned to the institutions for further refinement. Each institution’s dataset is represented using colored shapes to denote different cell types or datasets. Opaque shapes indicate data present within the institution, while semi-transparent shapes represent data that not in that institution but would be integrated if the data were centralized, offering a visual comparison between centralized Harmony and Federated Harmony.

Federated Harmony begins with the output embeddings generated by Federated Principal Component Analysis (Federated PCA) [24]. It operates through an iterative four-step process (Fig 1). First, each institution conducts local computation on its dataset, deriving summary statistics (e.g., centroids or row sum of some matrices) without sharing the raw data between institutions (Step ①). Next, these summary statistics are sent to a central server (Step ②), which is responsible for aggregating and updating the received statistics (Step ③). The central server then sends the aggregated summaries back to the institutions (Step ④), allowing each institution to adjust its local model using information from other institutions. Finally, each institution obtains the corrected and integrated embeddings. Fig 1 also compares the process of Federated Harmony and Harmony, and the contrast highlights the ability of Federated Harmony to borrow information from other institutions and correct data without sharing raw data, ensuring privacy while maintaining integration performance.

To evaluate the performance of Federated Harmony, we applied Federated Harmony to three types of datasets––scRNA-seq, spatial transcriptomics and scATAC-seq––and compared the results with those from Harmony. For Harmony, we followed the standard procedure. In contrast, Federated Harmony was tested through a simulation study where the dataset was split into distinct datasets based on their original batches, kept separate, and not integrated or shared, simulating real-world data privacy constraints. We then applied Federated Harmony to these split datasets to assess its effectiveness in integrating data in a decentralized manner. The Federated Harmony-integrated embeddings were then centralized solely for performance evaluation. All data source links can be found in S1 Table.

We first assess Federated Harmony on a scRNA-seq data. We use human peripheral blood mononuclear cells (PBMC) scRNA-seq data with 5 samples as an example. The whole blood was collected from four healthy donors, and scRNA-seq data were produced using the 10x Chromium platform. Furthermore, scRNA-seq data from an extra healthy donor from a pre-existing publicly available PBMC dataset was also included in the experiment. Within this group, samples 1 and 2 were sequenced together in one batch, while samples 3 and 4 were sequenced in a separate batch. The dataset for sample 5 was downloaded from a study previously conducted by 10x Genomics [25].

**Fig 2:**
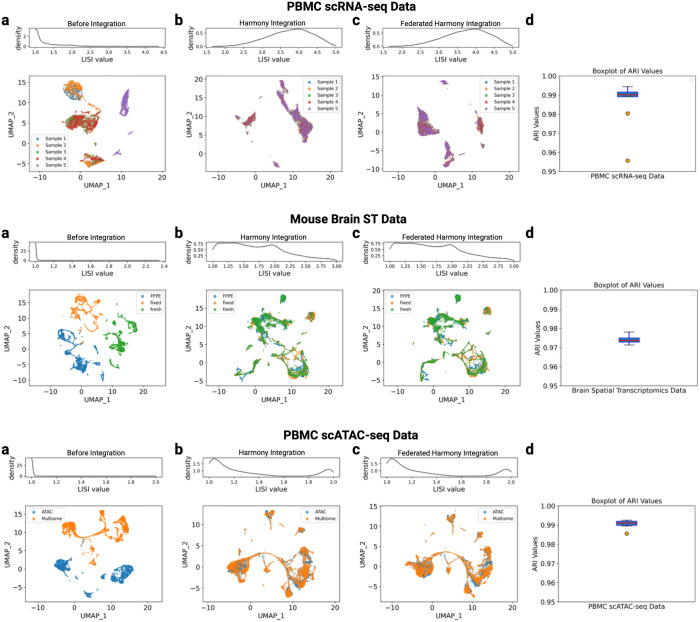
The performance of Federated Harmony on three types of single cell data. Each row represents results of a type of data. scRNA-seq data (first row); mouse brain tissue spatial transcriptomics data (second row); scATAC-seq data (third row). For **a-c**, the upper plot is the iLISI density plot, the lower one is the UMAP. **a**: iLISI density plot and UMAP before integration; **b**: iLISI density plot and UMAP after Harmony integration; **c**: iLISI density plot and UMAP after Federated Harmony integration; **d**: box plots of ARI values of naive k-means clustering results for Harmony-integrated and Federated Harmony-integrated embeddings.

Focusing on the PBMC scRNA-seq panels in Fig 2a–2c, the lower part plot shows UMAP [26] embeddings of PBMC scRNA-seq data in a two-dimensional space: before integration, after Harmony integration, and after Federated Harmony integration. Before integration, we applied a standard PCA pipeline followed by UMAP embedding. Fig 2a (UMAP Before Integration) for PBMC scRNA-seq data shows that samples 1 and 2 cluster together, as do samples 3 and 4, while sample 5 forms a separate cluster. This pattern indicates that the cells are grouped based on their original batches. We also tested our method on two additional scRNA-seq datasets, with similarly positive results. Detailed findings are provided in S1 Fig.

We then applied Harmony to integrate them and used the result as a baseline. As shown in Fig 2b (Harmony Integration) for PBMC scRNA-seq data, cells from all five samples are mixed. The UMAP generated from Federated Harmony-integrated embeddings closely resembles that of the Harmony-integrated embedding, shown in Fig 2c (Federated Harmony Integration) for PBMC scRNA-seq data. While the orientations of clusters within the UMAP plots may vary, such rotations or reflections do not affect the biological interpretation. This visual similarity indicates that Federated Harmony performs well for scRNA-seq data.

We then evaluated Federated Harmony on spatial transcriptomics (ST) data from 10X genomics. This dataset consists of three batches (FFPE, fixed, and fresh) of mouse brain tissue, with around 20,000 spatial spots in total, each corresponding to single cells. Before integration, cells are clustered in three distinct clusters by their batch as shown in the lower plot of Fig 2a (Before Integration) for brain ST data. After applying both Harmony and Federated Harmony, lower plots of Fig 2b (Harmony Integration) for brain ST data and Fig 2c (Federated Harmony Integration) for brain ST data show that the UMAP visualizations for the Federated Harmony-integrated and Harmony-integrated embeddings are highly similar, and both integrate cells from different batches together.

We further evaluated Federated Harmony on scATAC-seq data using two single-cell chromatin datasets derived from human PBMCs. One dataset was generated with the 10x Genomics multiome technology, providing both DNA accessibility and gene expression information for each cell. The other dataset was profiled using 10x Genomics scATAC-seq, containing only DNA accessibility data. The lower plot of Fig 2a (Before Integration) for PBMC scATAC-seq data shows a clear separation between the two batches. After applying both Federated Harmony and Harmony, as seen in lower plots of Fig 2b (Harmony Integration) for PBMC scATAC-seq data and Fig 2c (Federated Harmony Integration) for PBMC scATAC-seq data, cells from both datasets are well integrated, demonstrating the effectiveness on scATAC-seq data.

To compare the similarity between the centralized Federated Harmony- and Harmony-integrated embeddings, we examined the Adjusted Rand Index (ARI) from the naive k-means clustering results for both embeddings (the Federated Harmony-integrated embeddings were centralized here solely for performance evaluation). We varied the number of clusters (ranging from 2 to 10) in the k-means algorithm and calculated the ARI values for each scenario. The boxplots in Fig 2d named Boxplot of ARI values present ARI values, which consistently exceeded 0.95, regardless of cluster number. These high ARI values indicate almost identical integrated embeddings produced by both methods. To more rigorously assess integration quality, we computed the integration local inverse Simpson’s Index (iLISI) [8]. For each cell, iLISI measures the effective number of batches represented among its nearest neighbors. Values near 1 indicate single-batch neighborhoods (strong batch effect), whereas higher values indicate better mixing. As shown in Fig 2, the post-integration iLISI density curves for Harmony and Federated Harmony are highly similar across all types of data. Quantitatively, median iLISI improved as follows: PBMC scRNA-seq from 1.07 (Before Integration) to 3.79 (Harmony Integration) and 3.76 (Federated Harmony Integration); brain spatial transcriptomics from 1.00 to 1.63 and 1.64; scATAC-seq from 1.00 to 1.35 and 1.35. These results indicate that Federated Harmony achieves mixing comparable to centralized Harmony.

To evaluate scalability in a realistic setting, we used a published multi-omic blood cohort profiling patients with varying COVID-19 severity alongside influenza, sepsis, and healthy controls [27]. The resource includes matched immune measurements across modalities and reports severity-linked signatures spanning myeloid and lymphoid subsets, inflammatory mediators, and acute-phase responses. For our integration test we focused on the scRNA-seq data and selected 40 donors (treating each donor as a separate site/batch).

**Fig 3:**
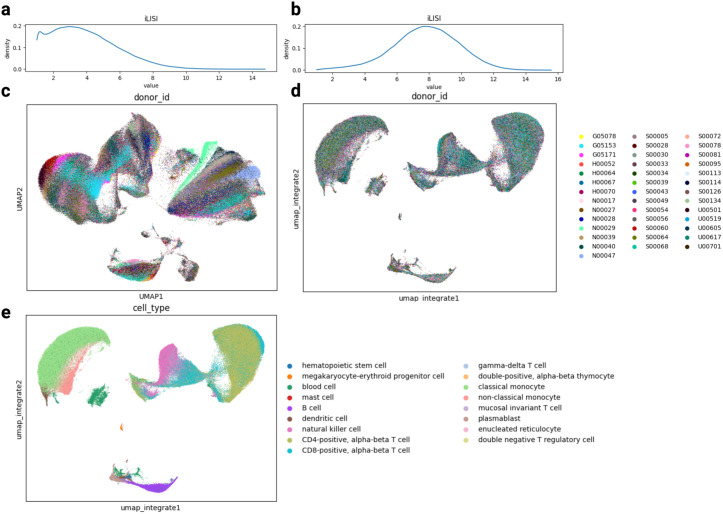
The results of Federated Harmony on large scale dataset. **a**: the iLISI density plot before integration; **b**: the iLISI density plot after Federated Harmony integration; **c**: UMAP before integration by donor; **d**: UMAP after Federated Harmony integration by donor; **e**: UMAP after Federated Harmony by cell type.

The results of Federated Harmony are shown in Fig 3. Before integration, the combined UMAP was dominated by donor identity in some regions with fragmented cell-type structure, which can be proven by the iLISI density map. For density map before integration, the density peaks around 1 – 4 and then tapers off and only a tiny tail exceeds 10, which indicates strong batch effects for a 40-batch data. After Federated Harmony, the embedding organized by cell type while the donors mixed across clusters. Quantitatively, median iLISI improved from 3.6 to 7.76. With the corrected embedding, each site can independently perform downstream analyses, such as cell type annotation. S3 Fig shows per-donor panels’ cell type annotation results.

### Computational efficiency, scalability and overhead

We also evaluated the computational efficiency of Federated Harmony and Harmony across different datasets, assessing running time (in seconds) and iterations required for convergence (S2 Fig). Federated Harmony consistently outperformed Harmony, requiring fewer iterations and shorter running times ideally. For instance, Federated Harmony converged in approximately 15 seconds and 10 iterations for PBMC scRNA-seq data (5 batches), while Harmony required 45 seconds and 25 iterations. Similarly, for brain spatial transcriptomics data (3 batches), Federated Harmony completed in 20 seconds with 9 iterations compared to Harmony’s 44 seconds and 15 iterations. The efficiency gap became more pronounced with an increasing number of batches, highlighting Federated Harmony’s scalability in multi-batch scenarios, where it offers a more computationally effective solution for large datasets. This improvement stems from two key factors. First, Federated Harmony performs some computations independently at each institute, parallelly distributing work that Harmony processes serially on a single server. Second, each federated round estimates batch-effect parameters from cluster-level averages, whose low variance allows the server to solve a closed-form weighted least squares problem and take larger steps toward the optimum compared to the small, per-cell adjustments used by Harmony (see Method). In our benchmarks this yields fewer global rounds to convergence.

Per institute, Federated Harmony only computes partial statistics in Harmony such as cluster centroids,co-occurrence matrix and intermediate values of correction matrix, so the cost in each institute is less than running Harmony once on the same data. For exchanging summary statistics between institutes and the center, only cluster-level sufficient statistics like centroids (*d* dimensions) and batch–cluster co-occurrence matrix (*K* clusters, *B* institutes) are transmitted, requiring no more than *Kd* or *KB* floats, whichever is larger. For example, with *d* = 30 PCs, *K* = 400 clusters, the centroids needed to be transmitted are approximately 12,000 floats (~48 KB float32). Thus, per-round transfer time (<0.01 s) and cost at 100 Mbps bandwidth is negligible in real-world application. Communication grows with *K* rather than the number of cells *N*_*b*_, the network cost remains modest provided clustering resolution tracks biological heterogeneity even if raw data size increases. Consequently, the workload for each institute fits easily on a standard workstation or laptop, and because each round transmits usually no more than 1 MB of summaries, the network overhead remains negligible on any stable internet connection. The workflow therefore can easily scale to collaborations with many participating institutes.

To report the full cost of the workflow, we also measured the total end-to-end time including Federated PCA in data preprocessing steps followed by Federated Harmony: on PBMC scRNA-seq (1,000 genes) Federated PCA took 25 s and Federated Harmony 15 s (total 40 s); on brain spatial transcriptomics (19,465 genes) Federated PCA took 688 s and Federated Harmony 20 s (total 708 s); on scATAC-seq (108,377 peaks) Federated PCA took 68 min and Federated Harmony 22 s (total **~**68 min). Empirically, Federated PCA time grows approximately linearly with the number of features and the number of samples. Note that all timings were obtained on a single workstation that emulates multiple institutes sequentially. Therefore, the reported wall-clock times are a conservative upper bound. In a real federated deployment, institutes compute in parallel, so per-round time is dominated by the slowest site plus a small communication term.

### Incomplete and heterogeneous sites

In federated biomedical applications, incomplete or heterogenous data among different site is a common issue. Federated Harmony follows Harmony’s assumption of a shared feature space, but it remains practical when deviations occur. When features are missing at some sites, we project each site onto the intersected set of highly variable genes (or another agreed feature subset) provided that this intersection still captures the key biology. If a site lacks a particular cell type, it contributes no statistics for that cluster; the parameters are updated from the remaining sites, which is analogous to how Harmony handles batches in which that cell type is absent. Cases in which sites measure different modalities (for example, scRNA-seq versus scATAC-seq or spatial data) are beyond the current scope. Potential extensions include block-wise updates that skip unavailable modalities or representation-learning layers that map heterogeneous modalities into a shared latent space.

### Privacy risk of sharing PCA-derived embeddings

Although principal-components are a convenient low-dimensional representation, they are not automatically outside the scope of data-protection law. Studies [28,29] have demonstrated that embeddings produced by PCA and other dimension reduction methods can be inverted to recover significant portions of the original high-dimensional data via a neural-network reconstruction attack, which means that sharing PCA embeddings carries a risk of raw data leakage or of inferring sensitive patient attributes. From a regulatory perspective, most frameworks including the EU GDPR (Recital 26; Art. 9), China’s PIPL (Arts. 4, 28, 73), and the U.S. HIPAA de-identification rule define personal or sensitive information by identifiability, not by data type. In practice this means that any per-sample PCA embedding remains regulated if it reveals or can be used to reveal patient health/genetic attributes. Together, these considerations indicate that PCA cannot be assumed safe to share in federated settings. To guard against emerging inversion and attribute-inference threats, Federated Harmony never shares embeddings; instead, it exchanges only minimal aggregated summary statistics (e.g., cluster centroids, co-occurrence counts), ensuring compliance with the strictest privacy requirements. We introduce a four-level privacy taxonomy (Table 1). The tiers progress from the most identifiable data—raw sequence reads (Tier 0)—through processed count matrices (Tier 1) and per-sample low-dimensional embeddings such as PCA embeddings (Tier 2), to the least identifiable representation, coarse aggregate statistics like cluster centroids and co-occurrence counts (Tier 3). This framework makes explicit the re-identification risk associated with each data type and motivates our decision to share only Tier 3 outputs in Federated Harmony.

**Table 1:** Privacy levels and information exchanged by Federated Harmony.

Tier	Example Data	Typical risk	Protection in Federated Harmony
**Level 0**	Raw reads	Direct identifiers & rare variant leakage	Never exchanged
**Level 1**	Count matrix, cell metadata	Gene- or sample-level linkage possible	Never exchanged
**Level 2**	PCA embeddings	Reconstruction/ attribute inference	Never exchanged
**Level 3**	Cluster centroids, co-occurrence counts, etc.	Minimal risk	Shared across sites

## Conclusion

In this study, we developed Federated Harmony, a data integration method for distributed single-cell multi-omics data that preserves data privacy. Our results demonstrate that Federated Harmony successfully integrates multi-institutional datasets, producing embeddings comparable to those of the standard Harmony approach. UMAP visualizations, iLISI and ARI values confirm that Federated Harmony effectively removes the non-biological variations in the dataset without requiring direct data sharing. Additionally, Federated Harmony can reduce integration time and iterations theoretically. This work fills a gap in the literature, as federated learning methods for single-cell data integration remain underexplored.

However, there are limitations to our study. Our method inherently involves multiple communication rounds between institutes, which could be a challenge in real-world applications like some other federated learning methods. Additionally, for preprocessing scATAC-seq data, TF-IDF normalization and Latent Semantic Indexing (LSI) are also commonly used, whereas our pipeline employs log-normalization and Federated PCA for this step. Finally, for preprocessing, we employed Federated PCA, which, though occasionally time-consuming, is external to our core method and could be optimized further in future implementations.

Future work should prioritize reducing communication rounds without compromising accuracy, as this will be critical for enhancing the applicability of Federated Harmony. Improving the computational efficiency of Federated PCA is another essential step toward broadening the method’s scalability. Additionally, optimizing the preprocessing steps for scATAC-seq data remains important to ensure consistency across data types. Once these advancements are achieved, the method can be tested on real-world applications, moving beyond simulations to validate its robustness in practical scenarios.

## Method

We first define all notation of variables we will involve in our method. The dimensionality of the embedding (for example, the number of PCs) is denoted as *d*; the number of institutions/batches is denoted as *B*; *N* is the number of total samples; *N*_*b*_ is the number of samples institutions/batch *b*; *K* is the number of clusters.

$\phi \in \{\text{0},\text{1}{\}}^{B\times N}$ The input one-hot assignment matrix of cells (columns) to batches/institutes (rows).
${Pr}_{b}\in [\text{0},\text{1}{]}^{B}$ Frequency of batches/institutes, i.e., $N_{b}/N$.
$R\in [\text{0},\text{1}{]}^{K\times N}$ The soft cluster assignment matrix of cells (columns) to clusters (rows). Each column is a probability distribution and thus sums to 1.
$O\in [\text{0},\text{1}{]}^{K\times B}$ The observed co-occurence matrix of cells in clusters (rows) and batches/institutes (columns).
$E\in [\text{0},\text{1}{]}^{K\times B}$ The expected co-occurence matrix of cells in clusters and batches, under the assumption of independence between cluster and batches/institutes assignment.
$Z^{\left(b\right)}\in R^{d\times N_{b}}$ The input embedding at institute *b*, to be corrected in Federated Harmony. The PCA embeddings of cells are often used.
$\hat{Z^{\left(b\right)}}\in R^{d\times N_{b}}$ The corrected embedding after batch correction at each institute, output by the proposed Federated Harmony.
$R^{\left(b\right)}\in [\text{0},\text{1}{]}^{K\times N_{b}}$ The soft cluster assignment matrix of cells (columns) to clusters (rows) at institute *b*. Each column is a probability distribution and thus sums to 1.
$Y\in R^{d\times K}$ Global cluster centroid locations.
$Y^{\left(b\right)}\in R^{d\times K}$ Updated cluster centroids at institute *b.*

### Harmony overview

The Harmony algorithm takes a PCA embedding of cells (*Z*) and their corresponding batch labels (ϕ) as inputs and produces a batch corrected embedding ($\hat{Z}$). It iterates between two stages: maximum diversity clustering and a mixture model based linear batch correction.

The objective function for maximum diversity clustering is defined by Eq. (1):


$$\underset{R,Y}{\text{min}}\sum_{i,k}R_{ki}|Z_{i}-Y_{k}{|}^{\text{2}}+\sigma R_{ki}\text{log}R_{ki}+\sigma \theta R_{ki}\text{log}\left(\frac{O_{ki}}{E_{ki}}\right)\phi_{i},$$

$$\text{s}.\text{t}.\forall_{\text{i}}\forall_{\text{k}}R_{ki}>\text{0},\forall_{\text{i}}\sum_{\text{k}=\text{1}}^{\text{K}}R_{ki}=\text{1},$$ (1)

where σ is a hyperparameter. The optimization of the objective function is shown in Eq. (2):


$$R_{ki}=\frac{{\left(\frac{O_{ki}}{E_{ki}}\right)}^{\text{2}}\text{exp}\left(-\frac{\text{2}\left(\text{1}-Y_{k}^{⊤}Z_{i}\right)}{\sigma }\right)}{\sum_{k=\text{1}}^{K}{\left(\frac{O_{ki}}{E_{ki}}\right)}^{\text{2}}\text{exp}\left(-\frac{\text{2}\left(\text{1}-Y_{k}^{⊤}Z_{i}\right)}{\sigma }\right)}.$$ (2)

The mixture of experts correction calculates a correction factor for each cluster *k* is defined in Eq. (3):


$$W_{k}={\left(\phi^{*}diag\left({\text{R}}_{\text{k}}\right)\phi^{-\text{T}}+\lambda \text{I}\right)}^{-\text{1}}\phi^{*}diag\left({\text{R}}_{\text{k}}\right){\text{Z}}^{\text{T}},$$ (3)

where $\phi^{*}\leftarrow \text{1}||\phi$, and then performs the batch correction by Eq. (4):


$$\hat{Z}=Z-W_{k}^{T}\phi^{*}diag\left(R_{k}\right).$$ (4)

### Federated Harmony overview

The steps of Federated Harmony fundamentally retain the procedure of Harmony, yet it makes a significant difference in execution by leveraging the federated computing principles-namely, local computations, central aggregation, and privacy preservation, as shown in Fig 1. At the beginning, each institute log-normalizes their dataset. Then, like the procedure of Harmony [8], Federated Harmony begins with local low dimensional embeddings of cells. Under the assumption that the data cannot be shared, traditional dimension reduction methods cannot be used since they presuppose the data are centrally aggregated. In response to these constraints, we used Federated PCA [24] at the preprocessing step, a method that enables each institutes to derive principal components for their local data by leveraging statistical summaries from data distributed across different institutes, without necessitating direct data sharing. The outcome of Federated PCA closely approximates the results when data from all institutes is first pooled together for PCA and then the corresponding principal components (PCs) are distributed back to their original locations.

**Fig 4:**
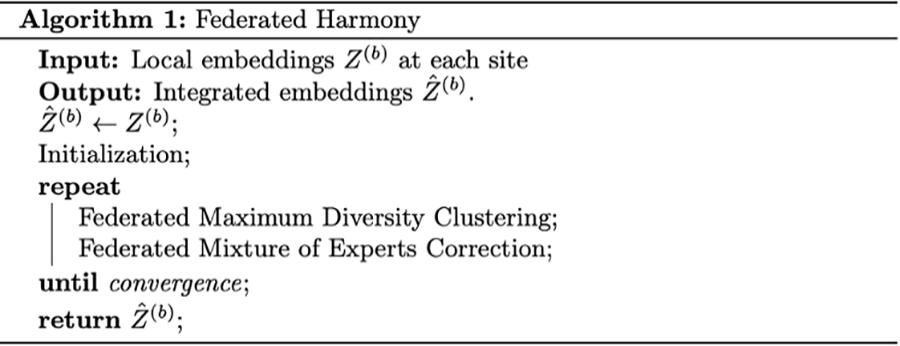
Federated Harmony Algorithm.

Federated Harmony (Algorithm in Fig 4) utilizes local embeddings and iterates between two stages, similar to Harmony. These stages include federated maximum diversity clustering and federated mixture model-based linear batch correction.

### Initialization

Federated Harmony first initialize the global centroids using Federated k-means [30]. By the definition of the soft cluster assignment matrix of cells (columns) to clusters (rows) *R* defined in Harmony [8], the *i*^*th*^ column in *R* corresponds to the probability distribution of the *i*^*th*^ cell across different clusters. This means that for the *i*^*th*^ cell, each entry in the column of *R* indicates the likelihood of that cell belonging to each cluster, i.e., $\sum_{k=\text{1}}^{K}R_{ki}=\text{1}$. The important aspect of calculating *R* is that its calculation for each column is based exclusively on the data corresponding to the individual cell, so *R* can also be represented as $R=[R^{\left(\text{1}\right)}R^{\left(\text{2}\right)}\ldots R^{\left(B\right)}]$ if there are *B* batches of data in the dataset and we order the cells by their original batch. Thus, each individual institution can independently initialize its own soft cluster assignment matrix $R^{\left(b\right)}$ by using the same strategy as in Harmony shown in Eq. (5):


$$R^{\left(b\right)}=\frac{exp\left(-\frac{\text{2}\cdot \left(\text{1}-Y^{T}\cdot Z^{\left(b\right)}\right)}{\sigma }\right)}{\sum_{i}exp\left(-\frac{\text{2}\cdot \left(\text{1}-Y^{T}\cdot {\text{Z}}^{\left(b\right)}\right)}{\sigma }\right)},$$ (5)

where $\Sigma_{i}$ denotes the column-wise sum.

Initialization of the matrix *O* is achieved through the operation $O\leftarrow R\phi^{T}$. Within the Federated Harmony framework, direct access to batch (institute) indicators is absent. To circumvent this, each institution is assigned a unique index to signify the batch to which each cell belongs. Consequently, the center constructs a matrix ϕ based on the institute index b∈[1,B] and the respective cell counts *N*_*b*_ for each institute *b*. The matrix ϕ is structured as follows, where each row corresponds to a distinct institute and the columns within a row are populated with ones to indicate the membership of cells to that institute, with the dimensionality of each segment determined by $N_{\text{1}},N_{\text{2}},\ldots ,N_{B}$, and each institution will also receive their own $\phi_{b}$ shown in Eq. (6):


$$\phi =\left[\phi_{\text{1}}\phi_{\text{2}}\cdots \phi_{B}\right]=[\begin{array}{cccc} \text{1}\cdots \text{1} & \text{0}\cdots \text{0} & \cdots & \text{0}\cdots \text{0}\\ \text{0}\cdots \text{0} & \text{1}\cdots \text{1} & \cdots & \text{0}\cdots \text{0}\\ \vdots & \vdots & \ddots & \vdots \\ \underset{N_{\text{1}}}{\underbrace{\text{0}\cdots \text{0}}} & \underset{N_{\text{2}}}{\underbrace{\text{0}\cdots \text{0}}} & \cdots & \underset{N_{B}}{\underbrace{\text{1}\cdots \text{1}}} \end{array}].$$ (6)

Given the representation of *R* as $R=[R^{\left(\text{1}\right)}R^{\left(\text{2}\right)}\ldots R^{\left(B\right)}]$, the matrix *O* is derived by computing $O\leftarrow R\phi^{T}$, which yields Eq. (7):


$$O_{k,b}=\sum_{i=\text{1}}^{N_{b}}R_{k,i}^{\left(b\right)}$$ (7)

This formulation implies that the center requires only the summation across rows of $R^{\left(b\right)}$ for each institute *b* to construct the matrix *O*. The reason why we do not share $R^{\left(b\right)}$ to the center will be explained later in this section. For initialization of *E* in Harmony, $E\leftarrow R1Pr_{b}^{T}$ where R1 equals to a vector that sums each row of *R* in Eq. (8),


$$R1={\left[\sum_{i=1}^{N}R_{1i}\;\;\;\;\sum_{i=1}^{N}R_{2i}\;\;\;\;\;\;\;\;...\;\;\;\;\;\;\;\sum_{i=1}^{N}R_{Ki}\right]}^{T}.$$ (8)

**Fig 5:**
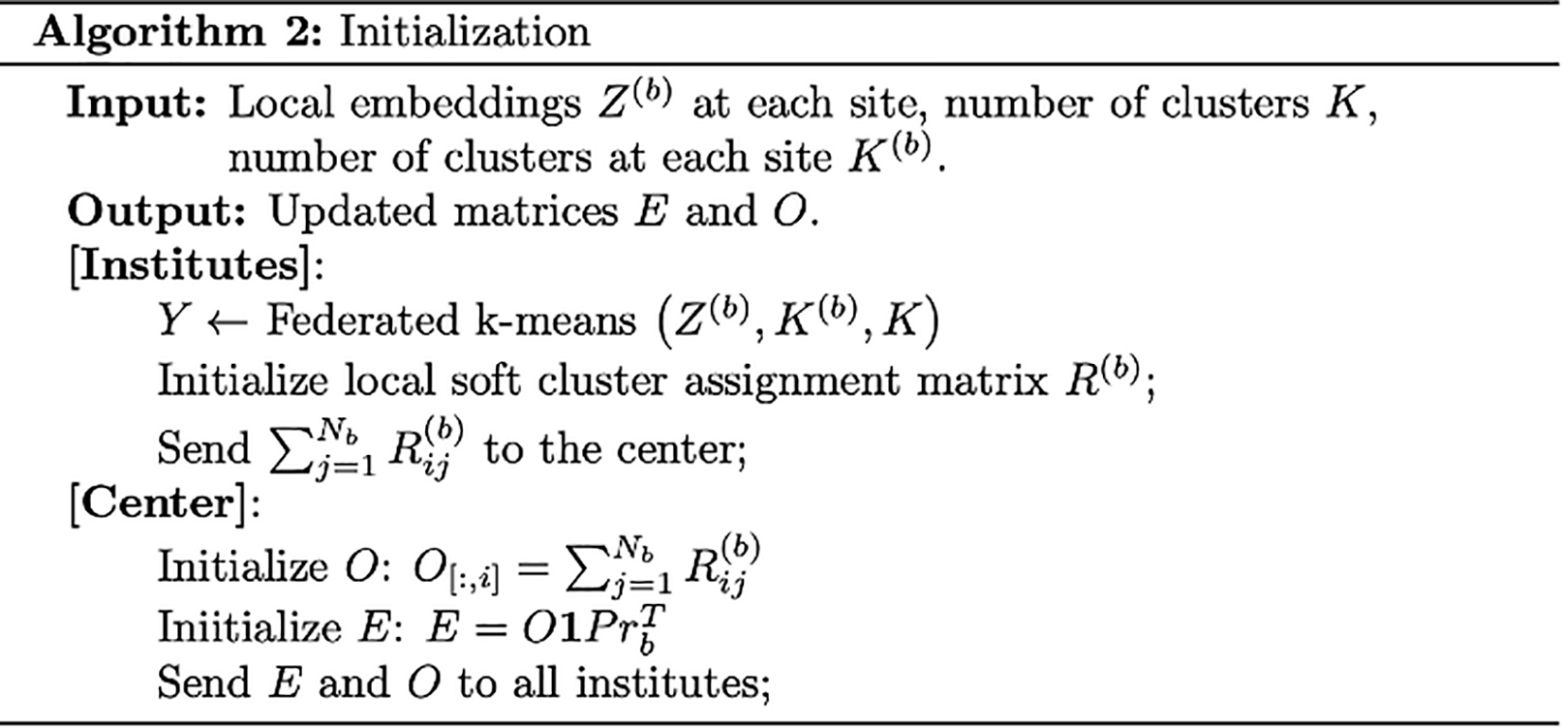
Initialization Algorithm.

In this case, R1 equals to the row sum of *O*, and thus $E=O1Pr_{b}^{T}$. In Federated Harmony settings, $Pr_{b}^{T}$ can be easily obtained by collecting the total number of cells in each institute. The initialization process is summarized in Algorithm in Fig 5:

### Federated maximum diversity clustering

After initializing $R^{\left(b\right)},E,$and *O*, the next step is to conduct a federated maximum diversity clustering. First, we update *Y* using the same strategy as in Harmony shown in Eq. (9):


$$Y=ZR^{T}=[\begin{array}{cccc} Z^{\left(\text{1}\right)} & Z^{\left(\text{2}\right)} & \ldots & Z^{\left(B\right)} \end{array}][\begin{array}{cccc} R^{(\text{1}{)}^{T}} & R^{({\text{2})}^{T}} & \ldots & R^{({B)}^{T}} \end{array}]=\Sigma_{b=\text{1}}^{B}Z^{\left(b\right)}R^{({b)}^{T}}.$$ (9)

This can be achieved by calculating $Y^{\left(b\right)}=Z^{\left(b\right)}R^{({b)}^{T}}$ at institute *b* and then the center aggregates and sums $Y^{\left(b\right)}$ to get *Y*. The equation here shows why we cannot directly share $R^{\left(b\right)}$, if $R^{\left(b\right)}$ is shared, the center can easily get the original embedding $Z^{\left(b\right)}$using $Z^{\left(b\right)}=Y^{\left(b\right)}{\left(R^{{\left(b\right)}^{T}}\right)}^{-\text{1}}$, which yields privacy concerns since embedding level data are shared.

In Harmony, the objective function for the update of *R* is optimized using block updates as the values of *O* and *E* change with *R*. Following the same idea, we also use block updates to update $R^{\left(b\right)}$. However, in Harmony, cells in each block are randomly selected, but in our method, we create blocks locally in each institution, which means each block will contain data from one batch. We call it sequential block update. Although we do not randomly assign cells to blocks across all data, there will be only small errors.

In the context of sequential block updates, the introduction of error can be analyzed through a detailed mathematical framework. Let the block size $n_{\text{block}}=\alpha N$, where 0<α≪1 (e.g., α = 0.05 for a block size of 5%). This implies the total number of blocks is $M=\frac{N}{n_{\text{block}}}=\frac{\text{1}}{\alpha }$.

In the sequential block update process, for each block *m*, the contributions of cells in that block are temporarily removed from matrices *O* and *E*, yielding modified matrices *O*_*m*_ and *E*_*m*_. The cluster assignments *R*_*m*_ for cells in block *m* are then updated using these modified matrices. Once the updates are made, the contributions of block *m* are re-added to *O* and *E* for subsequent block updates.

We define the error in the cluster assignment for block *m* as Eq. (10):


$$\delta R^{\left(m\right)}=R_{\text{sequential}}^{\left(m\right)}-R_{\text{ideal}}^{\left(m\right)},$$ (10)

where $R_{\text{sequential}}^{\left(m\right)}$ represents the cluster assignments from the sequential update, and $R_{\text{ideal}}^{\left(m\right)}$ represents the assignments under an ideal, simultaneous update. This error arises because $O^{\left(m\right)}$ and $E^{\left(m\right)}$ differ from their ideal values due to the exclusion of cells in block m.

When αis small, the proportion of excluded cells in each block is minimal, meaning that $O^{\left(m\right)}$ and $E^{\left(m\right)}$ deviate only slightly from their global values O and E. Using a first-order Taylor expansion, we approximate Eq. (11):


$$O^{\left(m\right)}=O-\Delta O^{\left(m\right)},\;E^{\left(m\right)}=E-\Delta E^{\left(m\right)},$$ (11)

where $\Delta O^{\left(m\right)}$ and $\Delta E^{\left(m\right)}$ represent the contributions of cells in block *m*. Since $\Delta O^{\left(m\right)}$ and $\Delta E^{\left(m\right)}$ are proportional to $n_{\text{block},}$, they are small when α is small.

For each cell *i* in block *m*, the diversity penalty $\Omega_{ki}$ is given by Eq. (12):


$$\Omega_{ki}=\theta \text{log}\left(\frac{O_{kb}^{\left(m\right)}+\text{1}}{E_{kb}^{\left(m\right)}+\text{1}}\right),$$ (12)

and the error in $\Omega_{ki}$ is defined in Eq. (13):


$$\delta \Omega_{ki}=\Omega_{ki}^{\text{sequential}}-\Omega_{ki}^{\text{ideal}}.$$ (13)

Using a first-order approximation, we get Eq. (14):


$$\delta \Omega_{ki}\approx \theta \left(\frac{\Delta E_{kb}^{\left(m\right)}-\Delta O_{kb}^{\left(m\right)}}{E_{kb}+\text{1}}\right),$$ (14)

where *E*_*kb*_ and *O*_*kb*_ are global counts, and $\Delta E_{kb}^{\left(m\right)}$ and $\Delta O_{kb}^{\left(m\right)}$ are small perturbations.

The update rule for *R*_*ki*_ is shown in Eq. (15):


$$R_{ki}\propto \text{exp}\left(-\frac{\text{2}\left(\text{1}-Y_{k}^{⊤}Z_{i}\right)}{\sigma }\right)\text{exp}\left(-\Omega_{ki}\right).$$ (15)

Thus, the error in *R*_*ki*_ is shown in Eq. (16):


$$\delta R_{ki}\propto R_{ki}^{\text{ideal}}\left(\text{exp}\left(-\delta \Omega_{ki}\right)-\text{1}\right)\approx -R_{ki}^{\text{ideal}}\delta \Omega_{ki}.$$ (16)

Assuming $\delta \Omega_{ki}$ is small, we approximate $\text{exp}\left(-\delta \Omega_{ki}\right)\approx \text{1}-\delta \Omega_{ki}$.

Since $\Delta O_{kb}^{\left(m\right)}$ and $\Delta E_{kb}^{\left(m\right)}$ are proportional to $n_{\text{block},}$ the error $\delta R_{ki}$ is also proportional to α, leading to a per-block error of *O* (α).

To quantify the cumulative error over all blocks, we sum the per-block errors as in Eq. (17):


$$\delta R=\sum_{m=\text{1}}^{M}\delta R^{\left(m\right)}.$$ (17)

With $M=\frac{\text{1}}{\alpha }$ blocks, the total error is bounded as in Eq. (18):


$$|\delta R|\leq M\times C\alpha =\frac{\text{1}}{\alpha }\times C\alpha =C.$$ (18)

Thus, the total error |δR| is bound by a constant *C* that is independent of α.

For the average error per cell, we have Eq. (19):


$$\text{Average}\left|\delta R_{ki}\right|=\frac{\left|\delta R\right|}{N}\leq \frac{C}{N}.$$ (19)

As *N* increases, the average error per cell decreases, indicating that the overall impact on clustering results is minimal. Additionally, the stochastic nature of the errors may lead to some cancellation over multiple blocks, further reducing the effect of the cumulative error on the clustering outcome.

**Fig 6:**
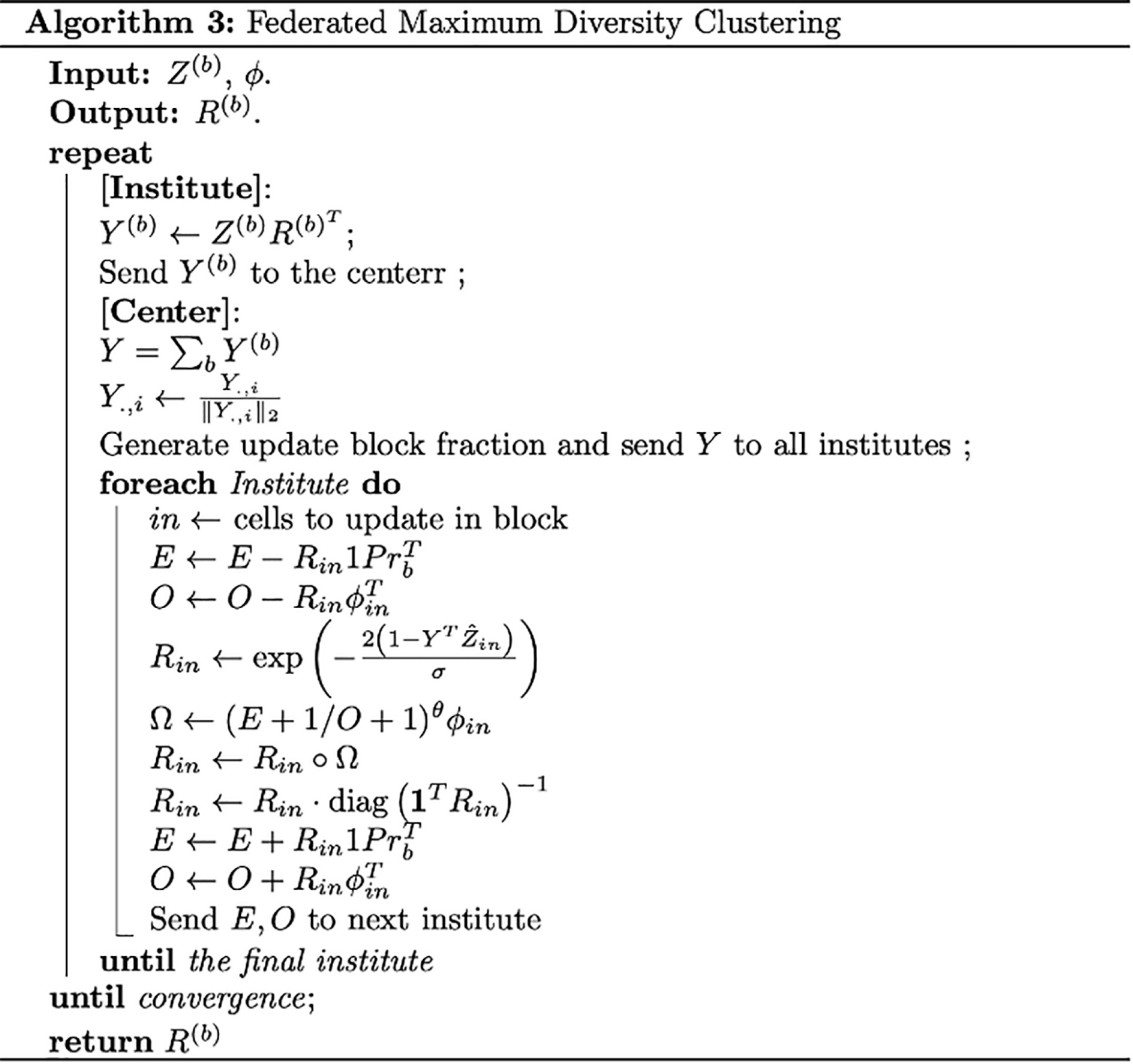
Federated Maximum Diversity Clustering Algorithm.

After each sequential block update in one institution, updated *O* and *E* are sent to the next institution for calculation until all institutions finish the update. After $R^{\left(b\right)}$ at each institute is updated, we repeat the process until convergence. Algorithm in Fig 6 shows the overview of federated maximum diversity clustering.

### Federated mixture of experts correction

The next step is to correct the embedding for each institute. In Harmony, the correction factors are calculated as $W_{k}={\left(\phi^{*}diag\left({\text{R}}_{\text{k}}\right)\phi^{*\text{T}}+\lambda \text{I}\right)}^{-\text{1}}\phi^{*}diag\left({\text{R}}_{\text{k}}\right){\text{Z}}^{\text{T}}$. In federated setting, the center can simply form a $\phi^{*}\leftarrow \text{1}||\phi$. If we order the cells by their batches, then the $\phi^{*}$ can be represented as: $\phi^{*}=\left[\phi_{\text{1}}^{*}\phi_{\text{2}}^{*}\cdots \phi_{B}^{*}\right]$ where $\phi_{b}^{*}={\left[1\phi_{b}\right]}^{\text{T}}\in R^{(B+\text{1})\times N_{b}}$, and $diag\left(R_{k}\right)$ can be represented as $diag\left(R_{k}\right)=diag\left(R_{k}^{\left(\text{1}\right)}R_{k}^{\left(\text{2}\right)}\cdots R_{k}^{\left(B\right)}\right)$.

Then we have Eqs (20) and (21)


$$\phi^{*}diag\left(R_{k}\right)Z^{T}=\sum_{b=\text{1}}^{B}\phi_{b}^{*}diag\left(R_{k}^{\left(b\right)}\right){Z^{\left(b\right)}}^{T},$$ (20)

$$\phi^{*}diag\left(R_{k}\right)\phi^{*T}=\sum_{b=\text{1}}^{B}\phi_{b}^{*}diag\left(R_{k}^{\left(b\right)}\right){\phi_{b}^{*}}^{T}.$$ (21)

For simplicity, we denote ${T_{k}=\phi }^{*}diag\left(R_{k}\right)Z^{T}$, ${\text{T}}_{\text{k}}^{\left(\text{b}\right)}=\phi_{b}^{*}diag\left(R_{k}^{\left(b\right)}\right){Z^{\left(b\right)}}^{T}$,$S_{k}=\phi^{*}diag\left(R_{k}\right)\phi^{*T}$ and ${S_{k}^{\left(b\right)}=\phi }_{b}^{*}diag\left(R_{k}^{\left(b\right)}\right){\phi_{b}^{*}}^{T}$. Both $T_{k}^{\left(b\right)}$ and $S_{k}^{\left(b\right)}$ can be computed locally at each institution, so to solve *W*_*k*_, we only need the center to aggregate these two terms from each institution. Once all *W*_*k*_ for k=1…k was calculated, we set $W_{k[\text{0},\cdot ]}$ to 0 to effectively remove batch-independent terms. Then ${\left\{W_{k}\right\}}_{k=\text{1}}^{K}$ are sent to each institution for data integration. In Harmony, the data are centralized and are corrected using Eq. (22)


$$\hat{Z}=\left[\hat{Z^{\text{1}}}\hat{Z^{\text{2}}}\cdots \hat{Z^{B}}\right]=Z-W_{k}^{T}\phi^{*}diag\left(R_{k}\right)=\left[Z^{\left(\text{1}\right)}Z^{\left(\text{2}\right)}\cdots Z^{\left(B\right)}\right]-W_{k}^{T}\left[\phi_{\text{1}}^{*}diag\left(R_{k}^{\left(\text{1}\right)}\right)\phi_{\text{2}}^{*}diag\left(R_{k}^{\left(\text{2}\right)}\right)\cdots \phi_{\text{2}}^{*}diag\left(R_{k}^{\left(B\right)}\right)\right],$$ (22)

but when the data are decentralized as in Eq. (23):


$$\hat{{\text{Z}}^{\text{b}}}=Z^{\left(b\right)}-W_{k}^{T}\phi_{b}^{*}diag\left(R_{k}^{\left(b\right)}\right).$$ (23)

**Fig 7:**
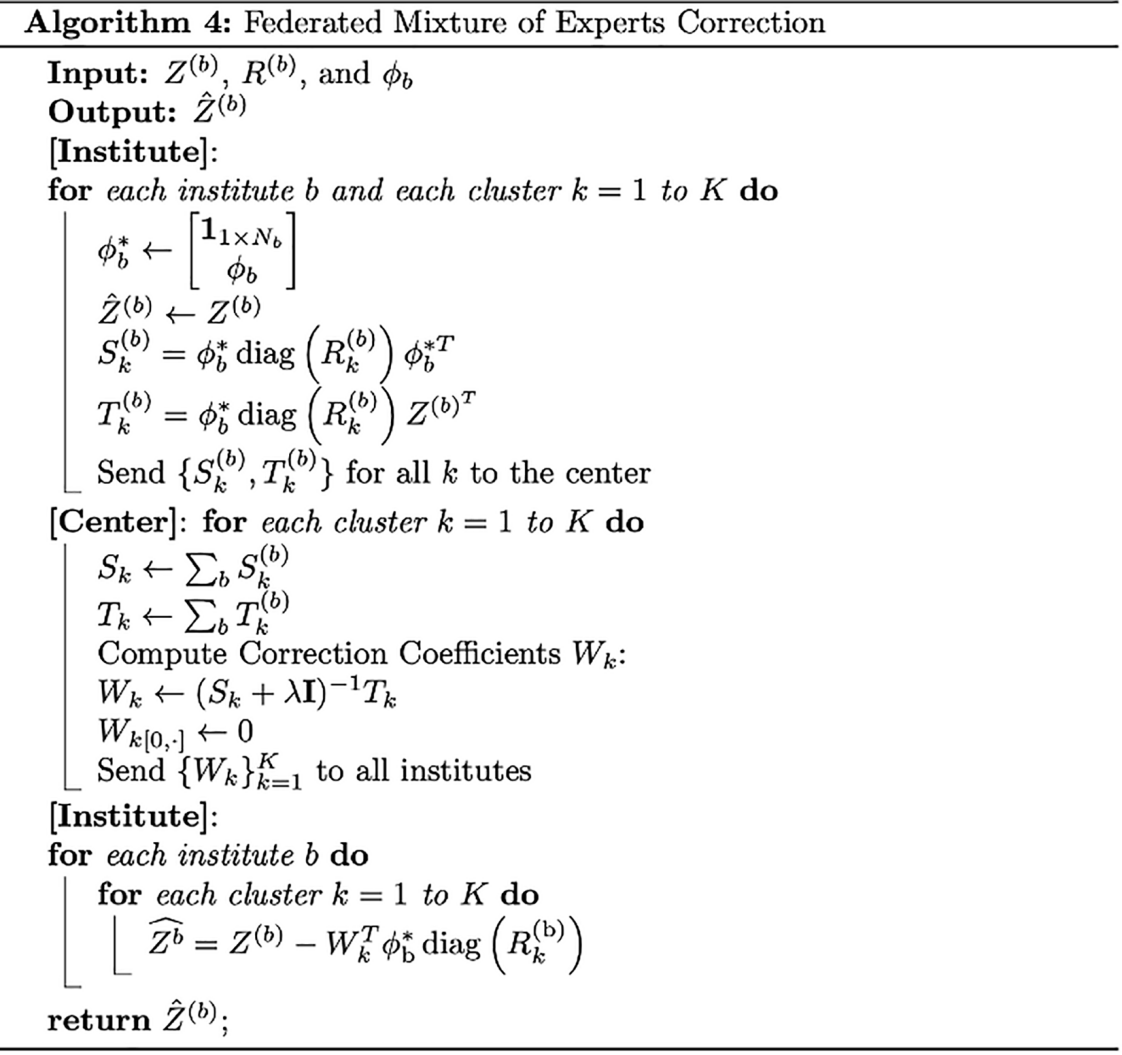
Federated Mixture of experts Correction Algorithm.

Each institution performs only matrix multiplication rather than engaging in compilated and time-consuming computations like matrix inverse. The whole process of federated mixture of experts correction can be summarized as Algorithm in Fig 7:

## Supporting information

S1 Fig The performance of Federated Harmony on two scRNA-seq data. For **a-c**, the upper plot is the iLISI density plot, the lower one is the UMAP. **a**: iLISI density plot and UMAP before integration; **b**: iLISI density plot and UMAP after Harmony integration; **c**: iLISI density plot and UMAP after Federated Harmony integration; **d**: box plots of ARI values of naive k-means clustering results for Harmony-integrated and Federated Harmony-integrated embeddings.
(TIF)

S2 Fig Comparison of running time and iterations for Federated Harmony and Harmony across different datasets and batch conditions. (TIF)

S3 Fig UMAPs by cell type for each donor (institution). Sample S00030 contains only 13 cells, which is why its corresponding subplot appears sparse.
(TIF)

S1 Info Further Result on scRNA-seq Data. (DOCX)

S2 Info Computational Efficiency Comparison. (DOCX)

S3 Info Per-donor (batch) Downstream Analysis. (DOCX)

S1 Table URL for datasets being used. (DOCX)
