# Supplementary figures and images for: Harmony-based data integration for distributed single-cell multi-omics data
Source: PLoS Comput Biol. 2025 Sep 30;21(9):e1013526. doi: 10.1371/journal.pcbi.1013526 (PMC12513639; doi:10.1371/journal.pcbi.1013526)

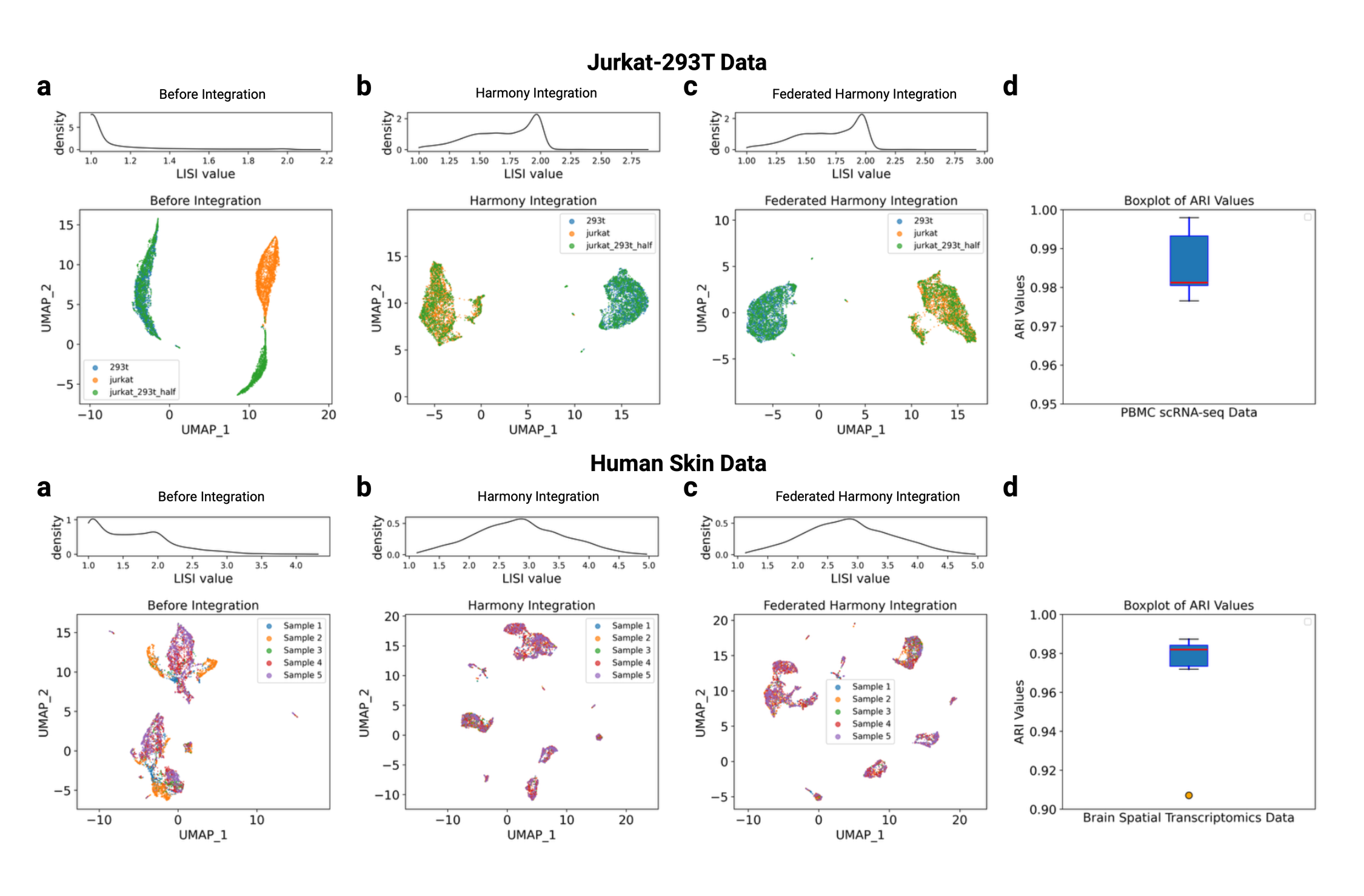

Supplement: S1 Fig — For a-c, the upper plot is the iLISI density plot, the lower one is the UMAP. a: iLISI density plot and UMAP before integration; b: iLISI density plot and UMAP after Harmony integration; c: iLISI density plot and UMAP after Federated Harmony integration; d: box plots of ARI values of naive k-means clustering results for Harmony-integrated and Federated Harmony-integrated embeddings. (TIF) [file pcbi.1013526.s001.tif]

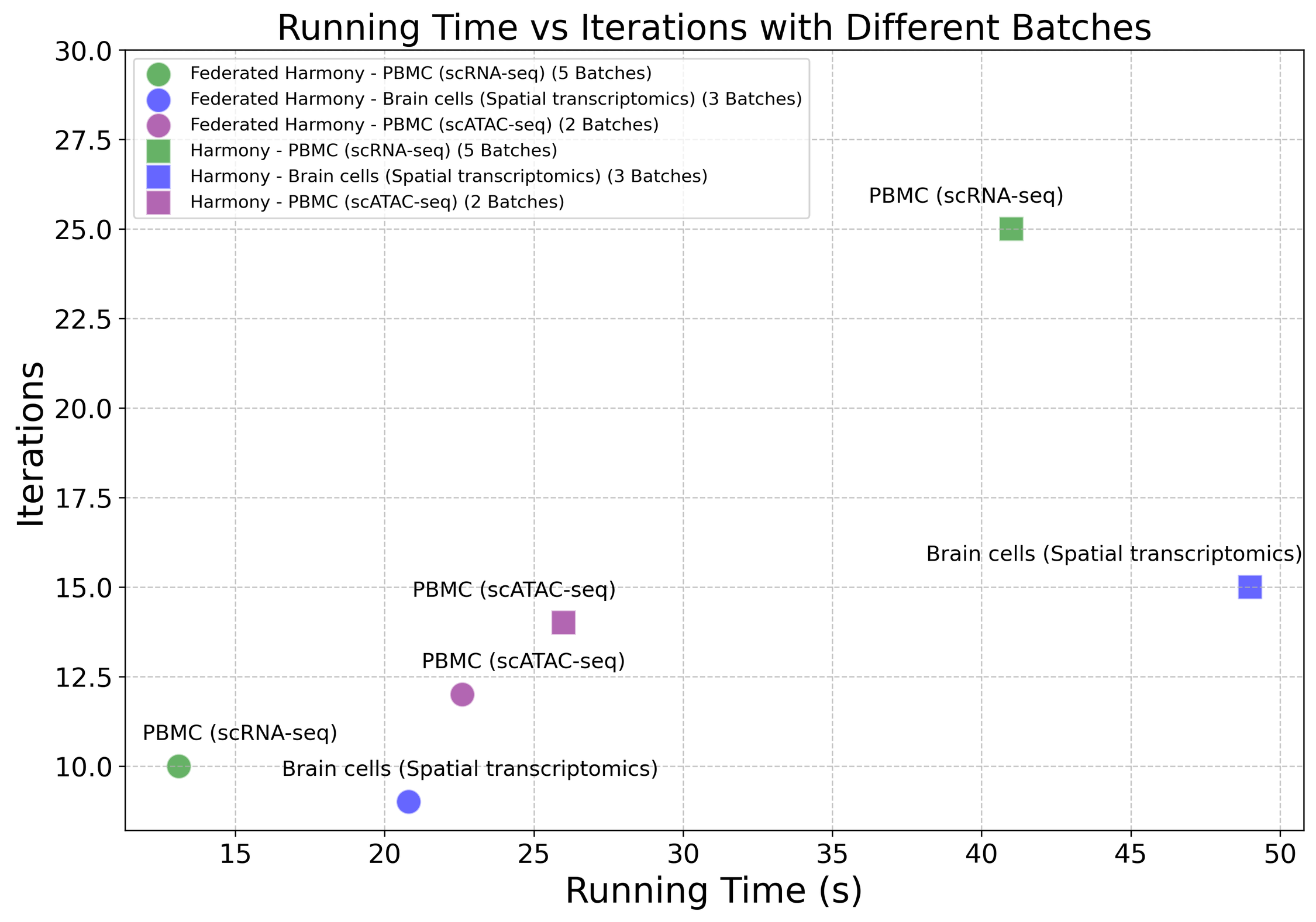

Supplement: S2 Fig — (TIF) [file pcbi.1013526.s002.tif]

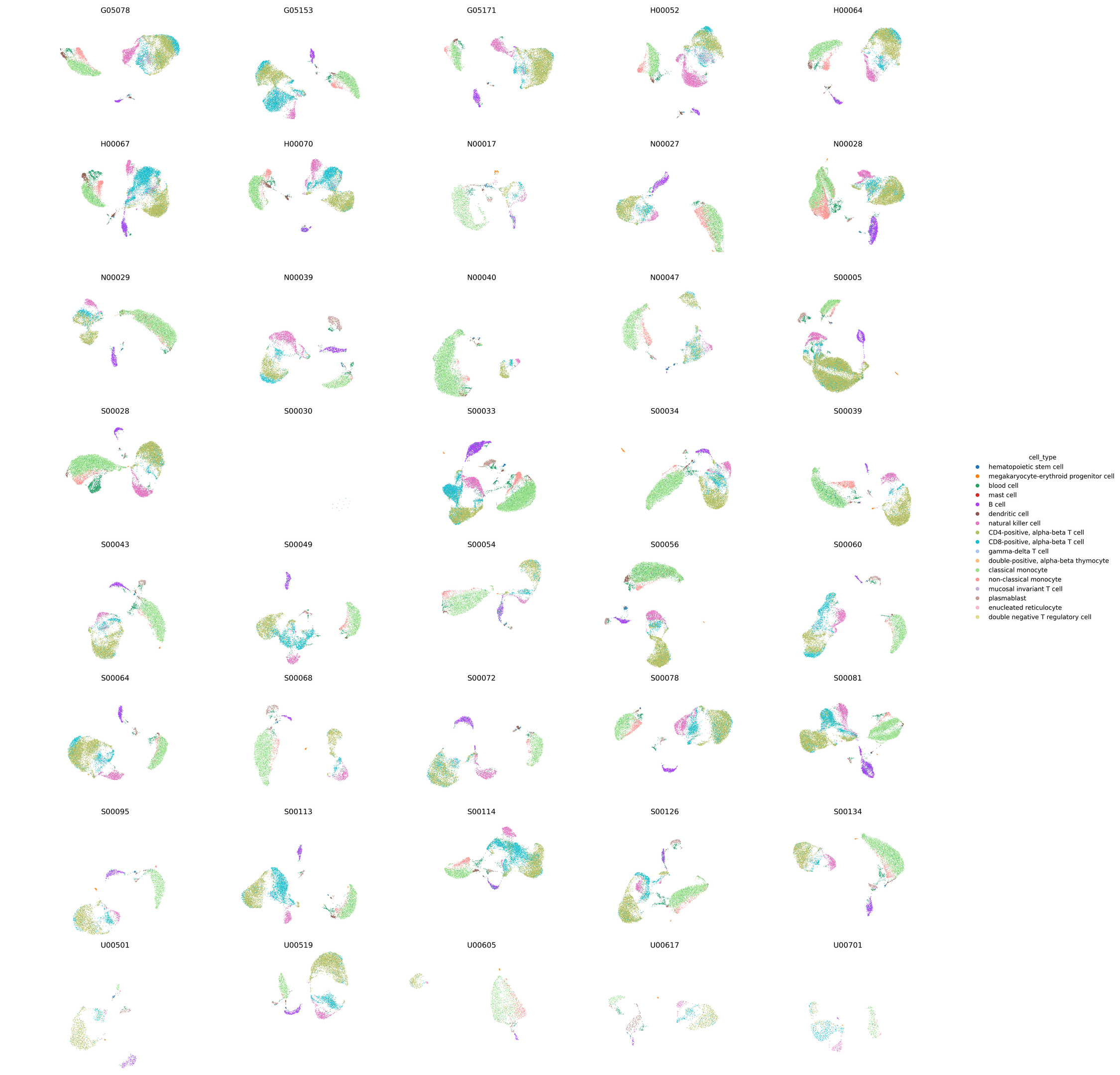

Supplement: S3 Fig — Sample S00030 contains only 13 cells, which is why its corresponding subplot appears sparse. (TIF) [file pcbi.1013526.s003.tif]
