# Supplementary material for: Harmony-based data integration for distributed single-cell multi-omics data
Source: PLoS Comput Biol. 2025 Sep 30;21(9):e1013526. doi: 10.1371/journal.pcbi.1013526 (PMC12513639; doi:10.1371/journal.pcbi.1013526)
Supplement: S1 Info — (DOCX) [file pcbi.1013526.s004.docx]

1. **Further Result on scRNA-seq Data**

Fig S1 shows the performance of Federated Harmony on two scRNA-seq datasets: (a) 293T-Jurkat and (b) Human Skin scRNA-seq data. The UMAP visualizations show the cell distribution before integration, after integration with Harmony, and after integration with Federated Harmony. In both cases, cells clustered by batch before integration, indicating strong batch effects. After applying both Harmony and Federated Harmony, these batch effects were successfully removed, with cells from different batches properly integrated. The Adjusted Rand Index (ARI) values, also presented in the figure, further demonstrate that Federated Harmony performs comparably to Harmony, achieving nearly identical clustering results while preserving data privacy. The median iLISI for Jurkat-293T data increases from 1 to 1.724 after Federated Harmony integration, compared to 1.721 after Harmony integration; For human skin data, the iLISI increases from 1.57 to 2.83 after Federated Harmony-integration, comparable to 2.833 after Harmony.
