# Supplementary material for: Harmony-based data integration for distributed single-cell multi-omics data
Source: PLoS Comput Biol. 2025 Sep 30;21(9):e1013526. doi: 10.1371/journal.pcbi.1013526 (PMC12513639; doi:10.1371/journal.pcbi.1013526)
Supplement: S2 Info — (DOCX) [file pcbi.1013526.s005.docx]

1. **Computational Efficiency Comparison**

We also compared the computational efficiency of Federated Harmony and Harmony using the above datasets. The computational efficiency was assessed based on the running time (in seconds) and the number of iterations required for convergence. For Federated Harmony, the total running time was calculated as the sum of the central server's computation time and the maximum local computation time among all institutions for each local computation round.

As shown in Fig S2, Federated Harmony generally demonstrates a more efficient performance compared to Harmony. For each data type, Federated Harmony consistently required fewer iterations and had shorter running times. For example, when processing the PBMC (scRNA-seq) data with 5 batches, Federated Harmony achieved convergence in approximately 15 seconds with 10 iterations, while Harmony took around 45 seconds and 25 iterations to converge.

Similarly, for the Brain cells (spatial transcriptomics) dataset with 3 batches, Federated Harmony completed its process in about 20 seconds with 9 iterations, compared to Harmony's 44 seconds and 15 iterations. The PBMC (scATAC-seq) dataset with 2 batches also followed this pattern, with Federated Harmony requiring about 22 seconds and 11 iterations, while Harmony took nearly 40 seconds and 14 iterations.

Federated Harmony achieves faster computation than traditional Harmony due to its distributed processing. Unlike Harmony, which relies on a centralized approach where all data must be aggregated and processed globally, Federated Harmony allows each institution only performs computation on a relatively **small local data**, the computational burden at each site is significantly reduced, leading to **faster processing times per iteration**. Additionally, because these computations occur **in parallel across multiple institutions**, Federated Harmony effectively leverages distributed computing resources, further accelerating the overall integration process.

For the number of iterations required to achieve convergency, in Harmony, because updates occur simultaneously across all batches, each iteration may only make small improvements before needing additional refinement, leading to a higher iteration count before convergence. However, in Federated Harmony, instead of updating all cells at once, updates occur in a sequential block-wise manner in institutions. Each institution processes its own data locally, and corrections are applied in an incremental way. This means that updates are introduced gradually, and each round of updates benefits from the refinements made in the previous step. Since errors are adjusted locally before moving to next institution, the system reaches an optimal solution in fewer steps compared to the global update strategy of traditional Harmony.

The results also indicate that as the number of batches increases, the efficiency gap between Federated Harmony and Harmony becomes more pronounced. Federated Harmony's convergence in fewer iterations and shorter running times, even with a larger number of batches (such as the 5-batch scRNA-seq dataset), demonstrates its scalability. In multi-batch scenarios where data integration can become computationally intensive, Federated Harmony offers a more effective solution, particularly when dealing with large datasets or numerous batches.
