# Supplementary material for: Harmony-based data integration for distributed single-cell multi-omics data
Source: PLoS Comput Biol. 2025 Sep 30;21(9):e1013526. doi: 10.1371/journal.pcbi.1013526 (PMC12513639; doi:10.1371/journal.pcbi.1013526)
Supplement: S3 Info — (DOCX) [file pcbi.1013526.s006.docx]

1. **Per-donor (batch) Downstream Analysis**

In Fig S3, Sample S00030 looks empty since the number of cells is only 13 and may be hard to visualize in the subplot. The cells are visible near the bottom right of the subplot.
