# Supplementary material for: Harmony-based data integration for distributed single-cell multi-omics data
Source: PLoS Comput Biol. 2025 Sep 30;21(9):e1013526. doi: 10.1371/journal.pcbi.1013526 (PMC12513639; doi:10.1371/journal.pcbi.1013526)
Supplement: S1 Table — (DOCX) [file pcbi.1013526.s007.docx]

| Analysis | Dataset | URL |
| --- | --- | --- |
| 293t-Jurkat scRNA-seq data | jurkat | <https://support.10xgenomics.com/single-cell-gene-expression/datasets/1.1.0/jurkat> |
|  | Hek293t | <https://support.10xgenomics.com/single-cell-gene-expression/datasets/1.1.0/293t> |
|  | Half | <https://support.10xgenomics.com/single-cell-gene-expression/datasets/1.1.0/jurkat:293t_50:50> |
| Human PBMC scRNA-seq data | Sample 1-4 | <https://www.ncbi.nlm.nih.gov/geo/query/acc.cgi?acc=GSE128066>. |
|  | Sample 5 | <https://support.10xgenomics.com/single-cell-gene-expression/datasets> |
| Human skin scRNA-seq data | Sample 1-5 | <https://www.ncbi.nlm.nih.gov/geo/query/acc.cgi?acc=GSE128066>. |
| Spatial transcriptomics data | FFPE | [https://www.10xgenomics.com/datasets/ffpe-visium-on-cytassist-mouse-brain-probe-based-whole-transcriptome-profiling-2-standard](https://nam12.safelinks.protection.outlook.com/?url=https%3A%2F%2Fwww.10xgenomics.com%2Fdatasets%2Fffpe-visium-on-cytassist-mouse-brain-probe-based-whole-transcriptome-profiling-2-standard&data=05%7C02%7CRUY45%40pitt.edu%7C44286c19ccb742bb6ec508dce3d24d68%7C9ef9f489e0a04eeb87cc3a526112fd0d%7C1%7C0%7C638635739582574048%7CUnknown%7CTWFpbGZsb3d8eyJWIjoiMC4wLjAwMDAiLCJQIjoiV2luMzIiLCJBTiI6Ik1haWwiLCJXVCI6Mn0%3D%7C0%7C%7C%7C&sdata=WDiBHVWLu%2Bafa3zM80K4UeGLuc8Pxx%2FRBUKaGvMxaLk%3D&reserved=0) |
|  | Fixed | [https://www.10xgenomics.com/datasets/fixed-frozen-visium-on-cytassist-mouse-brain-probe-based-whole-transcriptome-profiling-2-standard](https://nam12.safelinks.protection.outlook.com/?url=https%3A%2F%2Fwww.10xgenomics.com%2Fdatasets%2Ffixed-frozen-visium-on-cytassist-mouse-brain-probe-based-whole-transcriptome-profiling-2-standard&data=05%7C02%7CRUY45%40pitt.edu%7C44286c19ccb742bb6ec508dce3d24d68%7C9ef9f489e0a04eeb87cc3a526112fd0d%7C1%7C0%7C638635739582550774%7CUnknown%7CTWFpbGZsb3d8eyJWIjoiMC4wLjAwMDAiLCJQIjoiV2luMzIiLCJBTiI6Ik1haWwiLCJXVCI6Mn0%3D%7C0%7C%7C%7C&sdata=TFvjMPkThO%2F3J2kAljukHh1z44%2BItl5AGV%2FfhVCCZF8%3D&reserved=0) |
|  | Fresh | [https://www.10xgenomics.com/datasets/fresh-frozen-visium-on-cytassist-mouse-brain-probe-based-whole-transcriptome-profiling-2-standard-1](https://nam12.safelinks.protection.outlook.com/?url=https%3A%2F%2Fwww.10xgenomics.com%2Fdatasets%2Ffresh-frozen-visium-on-cytassist-mouse-brain-probe-based-whole-transcriptome-profiling-2-standard-1&data=05%7C02%7CRUY45%40pitt.edu%7C44286c19ccb742bb6ec508dce3d24d68%7C9ef9f489e0a04eeb87cc3a526112fd0d%7C1%7C0%7C638635739582595518%7CUnknown%7CTWFpbGZsb3d8eyJWIjoiMC4wLjAwMDAiLCJQIjoiV2luMzIiLCJBTiI6Ik1haWwiLCJXVCI6Mn0%3D%7C0%7C%7C%7C&sdata=91mgPwoT%2BRFkYbobR9txr2yYMatrRmM%2FKBWX%2BFke0q4%3D&reserved=0) |
| scATAC-seq data | Multiome | [https://cf.10xgenomics.com/samples/cell-arc/1.0.0/pbmc_granulocyte_sorted_10k/pbmc_granulocyte_sorted_10k_atac_fragments.tsv.gz](https://nam12.safelinks.protection.outlook.com/?url=https%3A%2F%2Fcf.10xgenomics.com%2Fsamples%2Fcell-arc%2F1.0.0%2Fpbmc_granulocyte_sorted_10k%2Fpbmc_granulocyte_sorted_10k_atac_fragments.tsv.gz&data=05%7C02%7CRUY45%40pitt.edu%7Cb0a693077e314c10f21c08dce3e66ed6%7C9ef9f489e0a04eeb87cc3a526112fd0d%7C1%7C0%7C638635826043147658%7CUnknown%7CTWFpbGZsb3d8eyJWIjoiMC4wLjAwMDAiLCJQIjoiV2luMzIiLCJBTiI6Ik1haWwiLCJXVCI6Mn0%3D%7C0%7C%7C%7C&sdata=mMWT5KKk8k0AI%2FPmdotT1icR2WW9%2FNLDQdiVCQdeuzc%3D&reserved=0) |
|  | ATAC | [http://cf.10xgenomics.com/samples/cell-atac/1.2.0/atac_pbmc_10k_nextgem/atac_pbmc_10k_nextgem_fragments.tsv.gz](https://nam12.safelinks.protection.outlook.com/?url=http%3A%2F%2Fcf.10xgenomics.com%2Fsamples%2Fcell-atac%2F1.2.0%2Fatac_pbmc_10k_nextgem%2Fatac_pbmc_10k_nextgem_fragments.tsv.gz&data=05%7C02%7CRUY45%40pitt.edu%7Cb0a693077e314c10f21c08dce3e66ed6%7C9ef9f489e0a04eeb87cc3a526112fd0d%7C1%7C0%7C638635826043179401%7CUnknown%7CTWFpbGZsb3d8eyJWIjoiMC4wLjAwMDAiLCJQIjoiV2luMzIiLCJBTiI6Ik1haWwiLCJXVCI6Mn0%3D%7C0%7C%7C%7C&sdata=SAiQeJxFTzvt06oihQUouEHAxIS0i3uSTC5%2FT%2BvLcEA%3D&reserved=0) |
| Large Scale scRNA-seq Data | Multi-omic blood cohort | https://explore.data.humancellatlas.org/projects/cdabcf0b-7602-4abf-9afb-3b410e545703 |
